# A Case of Geroderma Osteodysplasticum Syndrome: Unique Clinical Findings

**DOI:** 10.1055/s-0041-1740468

**Published:** 2021-12-17

**Authors:** Maha Alotaibi, Deema Aldhubaiban, Ahmed Alasmari, Leena Alotaibi

**Affiliations:** 1 Department of Genetics, Children's Hospital, King Saud Medical City, Riyadh, Saudi Arabia; 2 Department of Periodontology, King Saud Medical City, Riyadh, Saudi Arabia; 3 Department of Orthodontics, King Saud Medical City, Riyadh, Saudi Arabia; 4 Collage of Medicine, King Saud bin Abdul-Aziz University for Health Sciences, Riyadh, Saudi Arabia

**Keywords:** geroderma osteodysplasticum syndrome, lax skin, hyperextensible fingers, scoliosis, joints, abnormal

## Abstract

Geroderma osteodysplasticum (GO; MIM 231070) is characterized by a typical progeroid facial appearance, wrinkled, lax skin, joint laxity, skeletal abnormalities with variable degree of osteopenia, frequent fractures, scoliosis, bowed long bones, vertebral collapse, and hyperextensible fingers. The disorder results from mutations in the GORAB—golgin, RAB6 interacting. This gene encodes a member of the golgin family, a group of coiled-coil proteins on golgin that maps to chromosome 1q24. The encoded protein has a function in the secretory pathway, was identified by terminal kinase-like protein, and thus, it may function in mitosis. Mutations in this gene have been associated with GO. Herein, we describe the clinical presentation of one young male patient from related Saudi parents. Mutations, a homozygous frameshift mutation (c.306dup p.(pro 103 Thrfs*20)). Interestingly, phenotypic variability was observed in this patient with GO features that were more atypical than the cases reported in the literature as he looks tall stature where most of the cases reported were short and arachnodactyly fingers which mimic other syndromes.

## Introduction

Geroderma osteodysplasticum (GO; OMIM 231070) is a rare autosomal recessive disorder of the connective tissue and was delineated by Bamatter 1 in five members of a Swiss family. That family has been reviewed for more than 20 years. 2 3 Those patients had facial dysmorphism, hyperlaxity of skin facies looks a droopy, prematurely aged appearance, the eyelids and cheeks droop, the forehead is prominent, the nose is often prominent and fleshy, maxillary hypoplasia osseous changes, variable severity of osteoporosis, hyperextensible joints, kyphoscoliosis, bone fractures and vertebral collapse, and dental and congenital hip dislocation. The GO is clinically a distinct phenotype. 4 The disorder frequents in the Middle East and mainly in Oman. About 60 cases have been published to date. 5

The clinical phenotype of GO overlaps a heterogeneous group of disorders of the skin 6 including cutis laxa syndromes, autosomal dominant cutis laxa (MIM #123700), autosomal recessive cutis laxa (ARCLI; MIM #219100, ARCLII; MIM #219200, ARCLIII; MIM #219150), and wrinkly skin syndrome (MIM #278250). 7 And it is difficult to distinguish cutis laxa from GO. 8

The GO disorder is due to the mutation of a gene that encodes for a Golgi apparatus protein called *GORAB* . The physiological function of golgin, RAB6-interacting (GORAB) is poorly defined. The GORAB protein localizes to the Golgi apparatus and interacts with the small GTPase RAB6. 9 GORAB is important for vesicle transport at the Golgi complex and the correct processing of sugar chains on cargo proteins transiting through this compartment. Underlying defect of the skin and bone defects in GO patients is due to impaired coat protein complex I trafficking at the Golgi apparatus resulting in abnormal glycosylation of extracellular matrix composition.

## Clinical Report

**Fig. 1:**
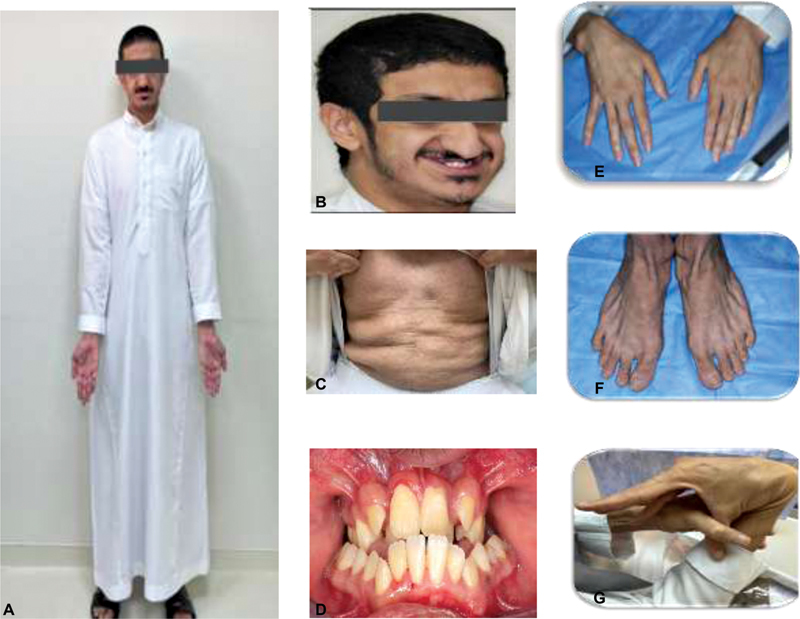
(A) Photograph of the patient showing tall stature, long face, arm span greater than height. (B) Typical facial features of patient include senile appearance, long and droopy face, mid-face hypoplasia, mandibular prognathism, large prominent, a fleshy tip of the nose and prominent ears, (C) wrinkly skin on the abdomen (D) showing generalized gingivitis and severe crowding, (E–G) showing hyperextensible fingers, arachnodactyly and wrinkly skin, prominent veins on the dorsum of the hands and the foot.

**Fig. 2:**
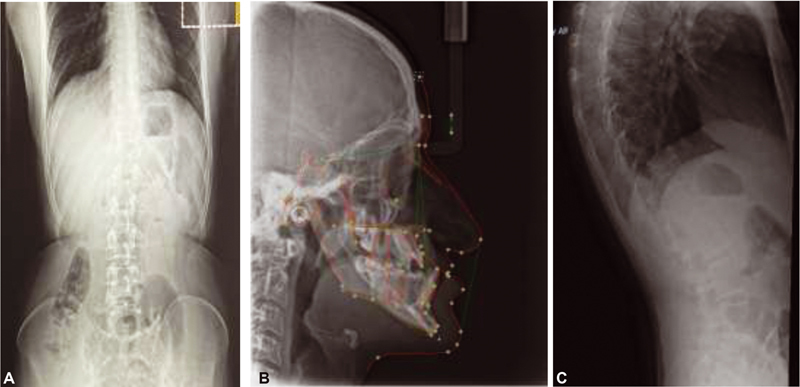
Radiological characteristics (A–C) radiographs of patient maintained thoracic kyphosis and lumbar lordosis. Diffuse decreased bone density; osteoporosis. Decreased height of T-7, 8, 9, and T-12 vertebral bodies. Normal height and alignment of the other vertebral bodies. C lateral cephalometric showed severe Cl III malocclusion due to combination between retrognathic maxilla and prognathic mandible.

This “a 24-year-old” Saudi man ( Fig. 1 ) was born at term via breech vaginal delivery after limited antenatal care to a primigravida mother. He is a product of consanguineous healthy parents and the family history was negative for congenital malformations or deaths. He was seen initially in the dental clinic with generalized gingivitis, gingival recession, and severe crowding ( Fig. 1 ) with dysmorphic facial features. The facial features consisted of progeroid appearance, long triangular face deep-set eyes, droopy cheek, mid-face hypoplasia, mandibular prognathism, large prominent, a fleshy tip of the nose and prominent ears, hyperextensible fingers, arachnodactyly fingers and toes with the appearance of wrinkly skin, and prominent veins in the dorsum of the hands, abdomen, and joint laxity. His height was 187.6 cm and weight was 73.4 kg. Her upper:lower segment ratio was 0.81 region. In addition, she had scoliosis with marked joint laxity. Radiological and skeletal findings support the diagnosis of GO radiographs ( Fig. 2 ). Dual-energy X-ray absorptiometry scan showed a decrease in bone density (total lumbar spine [L1–L4] Z score −3.1; total right femur Z score −2.5). There was no history of fracture and no recent hospitalization. He is a college student with good performance, normal intelligence.

## Discussion

GO was first described by Bamatter 1 in five members of a Swiss family and he referred them as Walt Disney dwarfs. It is inherited as an autosomal recessive disorder caused by mutations in the *GORAB* gene on chromosome 1q24, which encodes a protein important for Golgi-related transport. 10

The characteristics features of GO include the skin changes suggestive of premature aging, and various skeletal changes including osteoporosis, vertebral deformity, and fractures. The patient described here has old age facial appearance tall stature, all of the cases which have been reported were normal or short stature 2 9 11 wrinkled skin, mainly over the dorsum of the hand, laxity joint, and he had osteoporosis, kyphoscoliosis, a reduced upper/lower segment ratio, no history of fractures, and severe periodontal disease including generalized gingivitis, gingival recession, and severe crowding. With normal intelligence, even there were patients reported with GO to be mentally retarded. 11 Indeed, whole-exome sequencing was done for the patient, and written consent was obtained from the patient for the analysis. A blood sample in EDTA was collected from the patient , and written informed consent was obtained from the patient.

Genomic DNA is enzymatically fragmented, and target regions are enriched using DNA capture probes. These regions include ∼41 Mb of the human coding exome (targeting > 98% of the coding RefSeq from the human genome build GRCh37/hg19), as well as the mitochondrial genome. The generated library is sequenced on an Illumina platform to obtain at least 20x coverage depth for > 98% of the targeted bases. An in-house bioinformatics pipeline, including reading alignment to GRCh37/hg19 genome assembly and revised Cambridge Reference Sequence of the human mitochondrial DNA (NC_012920), variant calling, annotation, and comprehensive variant filtering is applied. All variants with minor allele frequency of less than 1% in Genome Aggregation Database and disease-causing variants reported in HGMD, in ClinVar, or CentoMD are evaluated. The investigation for relevant variants is focused on coding exons and flanking ± 10 intronic nucleotides of genes with clear gene-phenotype evidence (based on OMIM information).

It revealed the presence of a homozygous frameshift mutation (NM_152281.2:c.306dup). The GORAB variant c.306dup p.(Pro103Thrfs*20) introduces a premature stop codon that leads to the creation of a truncated protein. According to HGMD Professional 2020.3, this variant has previously been described in several patients/families with GO (PMID: 19681135, 27023906, 29620724). 12 It is classified as likely pathogenic (class 2) according to the recommendations of CENTOGENE and ACMG.

To date, 19 different GORAB mutations associated with geroderma osteodysplasticum (GO) syndrome have now been documented including seven missense/nonsense, one splicing, two small deletions, one small insertion, one small indel, and one gross deletion ( Table 1 ). These mutations had been reported from GO families from Saudi, Germany, Libya, Canada, Pakistan, Mexico, Italy, United States, and Azerbaijan. 5 10 13 In the reported GO gene mutations, more than one family from a common geographical area has been identified. These observations highlight that those mutations are ancestral and point to a distinct founder effect.

**Table 1:** GORAB mutations in different ethnic groups causing GO syndrome

Mutation	Ethnicity	Nucleotide change	Reference
Missense	Saudi	658G > C, p.A220P)	11
Nonsense	Germany	136G > T p Glu46X)	12
Nonsense	Libya	190C4T	12
Nonsense	Canada	367G4T	12
Nonsense	Mexico	367G4T	12
Nonsense	Italy	739C4T	12
Nonsense	United State	784C4T	12
Splicing	United State	662 + 5G4C	12
Small deletion	Germany	1050_1053delTCTT	12
Small insertion	Saudi	226 227 insA	11
Small indels	Pakistan	–1_1GA4CT	12
Gross deletion	Azerbaijan	Breakpoint between chr1:164037509–170654598 bp on chr 1q23.2-q24.2	14

Abbreviations: GO, geroderma osteodysplasticum; GORAB, golgin, RAB6-interacting.

## Conclusion

A differential diagnosis of GO with clinical phenotype overlap with other congenital disorder such as cutis laxa, Ehlers–Danlos' syndrome, Costello's syndrome, and the progeroid syndromes. 14 In conclusion, we report a GO patient with homozygous GORAB mutations with different clinical features that have not been reported before as tall stature and arachnodactyly and crowded teeth. The unusual finding and distinctive clinical phenotype of GO emphasize the usefulness of advance molecular genetic test for diagnosis.
